# DNA amplification tests at universal pre-admission screening with enhanced precaution strategies for asymptomatic patients with COVID-19

**DOI:** 10.1016/j.ijregi.2022.12.010

**Published:** 2022-12-31

**Authors:** Itaru Nakamura, Yusuke Watanabe, Hiroaki Fujita, Takehito Kobayashi, Hidehiro Watanabe, Takao Itoi

**Affiliations:** a Department of Infection Prevention and Control, Tokyo Medical University Hospital, Tokyo, Japan; b Department of Gastroenterology and Hepatology, Tokyo Medical University Hospital, Tokyo, Japan

**Keywords:** COVID-19, NAATs, Universal screening, Admission

## Abstract

**Objective:**

To analyse the effect of hospital pre-admission screening and enhanced precaution strategies on the transmission of severe acute respiratory syndrome coronavirus-2.

**Methods:**

This retrospective cohort study was conducted over 17 months from 11 May 2020 to 30 September 2021 at a large hospital in Tokyo. Universal DNA amplification tests were conducted during pre-admission screening, and enhanced precaution strategies were implemented for all patients with negative admission tests. The primary outcome was the occurrence of symptomatic coronavirus disease 2019 (COVID-19) in patients after admission. The secondary outcomes were time-series analyses of monthly positive admission test numbers, positive rates, clinical features in positive cases, and clinically confirmed nosocomial transmission.

**Results:**

In total, 32,081 patients were screened pre-admission (29,556 asymptomatic patients and 2525 symptomatic patients). Of the asymptomatic patients, 0.11% (*n*=32) tested positive and were admitted to a designated COVID-19 ward or were not admitted. Among the five inpatients who developed symptomatic COVID-19 during hospitalization, only two cases were related to a single nosocomial transmission.

**Conclusion:**

Pre-admission test screening was effective in identifying asymptomatic cases of COVID-19. This allowed administrators to quarantine patients or delay hospital admission. The combination of testing and enhanced precaution strategies for asymptomatic cases of COVID-19 may minimize nosocomial transmission.

## Highlights

• Universal admission screening and enhanced infection precautions were adopted.
• This was a retrospective study in Tokyo, from 11/5/2020 to 30 /9/2021.
• The positive rate in patients with asymptomatic coronavirus disease 2019 (COVID-19) was 0.11% (32 in 29,556).
• Five inpatients developed COVID-19 and nosocomial transmission occurred.
• Enhanced infection precautions may improve the defects of test screening.

## Introduction

As a strategy to prevent nosocomial transmission of severe acute respiratory syndrome coronavirus-2 (SARS-CoV-2), symptom-based admission screening, such as symptom interviewing, has been implemented in hospital settings [1]. Universal hospital admission screening using DNA amplification tests has also been reported as a strategy to identify asymptomatic cases of coronavirus disease 2019 (COVID-19), especially before the emergence of the Alpha variant [2], [3], [4], [5], [6], [7], [8]. However, as reported previously [2], the positive rate of such testing is typically small. The effectiveness of universal DNA amplification tests varied depending on the incidence of COVID-19 in the area during the study period. Therefore, the usefulness of test-based admission screening remains controversial. Furthermore, some authors have noted that screening using universal DNA amplification tests may not be cost-effective, despite the expectation of risk reduction. Laboratory-test-dependent admission screening can misdiagnose a presymptomatic patient or latent patient during the incubation period, as DNA amplification tests may be negative based on the natural features of COVID-19. In cases with negative DNA amplification test results, healthcare workers may underestimate the risk of infection and take less care [9]. Many failures to prevent nosocomial transmission have been suggested to originate from negative nucleic acid amplification test (NAAT) results at the asymptomatic or presymptomatic stage for patients with COVID-19.

Furthermore, a previous study reported the insufficient effectiveness of admission testing alone [9]. To address this issue, both adapted admission DNA amplification tests and enhanced infection precautions were used for all inpatients, even those with negative admission test results. Additionally, most studies of test-based admission screenings were conducted over short periods from a few weeks to a few months during the Wuhan clone pandemic phase, before the emergence of the Alpha variant. Japan faced five epidemic surges related to the Wuhan clone, the Alpha variant and the Delta variant. Therefore, this study analysed the effect of universal DNA amplification test admission screening and a combined infection prevention strategy over a long period (17 months) in a fluctuating high-prevalence setting with three COVID-19 variants before the emergence of the Omicron variant.

## Materials and methods

This retrospective observational cohort study was conducted to evaluate the effect of pre-admission universal DNA amplification tests for all inpatients and the implementation of enhanced infection precaution strategies during hospitalization. The study was conducted at Tokyo Medical University Hospital (TMUH), a 905-bed tertiary hospital, over 17 months from 11 May 2020 to 30 September 2021. Five waves of COVID-19 occurred in Tokyo during the study period: (a) the Wuhan clone from March 2020 to May 2020; (b) the Wuhan clone from July 2020 to September 2020; (c) the Wuhan clone from November 2020 to February 2021; (d) the Alpha variant from April 2021 to June 2021; and (e) the Delta variant from July 2021 to September 2021.

TMUH is located in Shinjuku City, the largest urban centre in Tokyo. The admission rate of TMUH averages more than 26,000 patients per year. Patients with a positive SARS-CoV-2 status are admitted to a designated hospital ward in TMUH, whereas those with a laboratory-confirmed negative test are admitted to the general hospital wards. Regardless of the hospital ward, all patients are admitted to equal pressure rooms, except for haematology patients who require positive pressure rooms. Only patients with COVID-19 requiring intensive care or aerosol-generating procedures are admitted to negative pressure rooms. The standard room designs are single-patient rooms or four-patient rooms without side rooms in both the designated COVID-19 ward and general wards. During the study, the standard room air exchange rate was two times per hour. All patients admitted during the study period were classified retrospectively as symptomatic or asymptomatic.

Vaccination against SARS-CoV-2 started in Japan on 17 February 2021, with healthcare workers prioritized over the general population. Vaccination rates for first and second doses were 88.69 million (70%) and 75.04 million (59.3%), respectively, at the end of the study period. For individuals aged ≥65 years, who account for the largest number of hospital admissions, the vaccination rates were 32.35 million (90.5%) for the first dose and 31.9 million (89.2%) for the second dose.

First, universal DNA amplification test screening was performed for all patients on admission, regardless of symptoms, to identify all patients with laboratory-confirmed SARS-CoV-2 infection. DNA amplification testing occurred within a maximum of 14 days before admission (mean 3 days), as 14 days was the maximum incubation period for all three variants. Additionally, after admission, if the patient became symptomatic or was identified as being at risk through contact tracing, retests were performed. When a patient was diagnosed with COVID-19 after admission, interviews and contact tracing were performed to judge the risk of transmission between patients and from patients to medical staff. Similarly, when medical staff developed COVID-19, they were surveyed about their work status, personal protective equipment compliance, patient contact, and social activity outside of the hospital to determine the risk of transmission from patients to medical staff or from medical staff to patients.

Planned admission patients underwent reverse transcription polymerase chain reaction (RT-PCR) assay (Cobas, Roche Molecular Systems, Branchburg, NJ, USA), which has 85% test sensitivity and 95% test specificity. The loop-mediated isothermal amplification (LAMP) test (Loopamp EXIA, Eiken, Taito-ku, Tokyo, Japan), which has 80% test sensitivity and 95% test specificity, was used for emergency admission patients. The thresholds for positive or negative results were based on the manufacturers’ manuals. If the LAMP test showed equivocal results, an RT-PCR assay was performed for confirmation.

Second, in all general wards, enhanced infection precaution strategies were implemented in addition to universal NAAT admission screening. The details of the enhanced infection precaution strategies are shown in Table 1. These infection precaution strategies during hospitalization were designed under the assumption that some patients may test positive following a negative admission test, in order to mitigate the spread to other inpatients. Highly test-dependent infection control strategies, such as a same-day admission test for all patients or retesting of all patients 4–7 days after admission, were not suitable at TMUH due to test capacity and cost. Thus, instead of highly test-dependent strategies, enhanced infection control strategies were adopted in this study.

**Table 1:** Enhanced infection precaution strategies with universal nucleic acid amplification test (NAAT) admission screening for severe acute respiratory syndrome coronavirus-2.

	Strategy
1	Universal surgical mask policy for all inpatients and healthcare workers regardless of symptomatic or asymptomatic status
2	Eye protection as a standard precaution for all high-risk care, such as aspiration, intubation and endoscopy, even for asymptomatic patients
3	Further NAATs for patients with unexplained fever or respiratory symptoms after admission testing
4	Family visit restrictions in the hospital
5	Restrictions on patients going outside during hospitalization
6	Symptom-based staff screening before the workday; self-isolation and completion of NAATs or any antigen test if symptomatic
7	Contact tracing and quarantine of identified persons

The primary outcome of the study was defined as the occurrence of laboratory-confirmed and symptomatic COVID-19 in patients after admission with negative admission screening tests. The secondary outcomes were defined as the total number of positive admission tests, time-series analyses of monthly positive admission tests, positive universal admission tests, positive admission tests, clinical features of positive admission cases, and the details of clinically confirmed nosocomial transmission of SARS-CoV-2 between patients and healthcare workers.

Additionally, a time-series comparison was performed between positive cases at admission screening at TMUH and COVID-19 notification trends in the whole Tokyo metropolitan area. The COVID-19 notification case numbers in the Tokyo metropolitan area were extracted from official data on the Tokyo Metropolitan Government website (https://stopcovid19.metro.tokyo.lg.jp/).

## Results

In total, 32,081 DNA amplification pre-admission tests were performed at TMUH during the study period. Among the tested patients, 29,556 were asymptomatic and 2525 were symptomatic on admission. Among the 29,556 patients who were asymptomatic on admission, 32 received a positive DNA amplification test result and were admitted to a designated COVID-19 ward or were not admitted. The positive rate among the asymptomatic cases was 0.11% (*n*=32). Among the 2525 inpatients who were symptomatic on admission, 334 received positive DNA amplification test results, resulting in a positive test rate of 13.2% (*n*=334).

Whereas 10 of the 32 asymptomatic patients with positive test results (31.3%) remained totally asymptomatic, five (15.6%) developed symptomatic COVID-19 after admission. Twelve patients (37.5%) reported being asymptomatic but showed incidental pneumonia on chest X-ray or computed tomography. This meant they had COVID-19 but did not recognize the symptoms (self-unrecognized patients). Five patients (15.6%) had a previous history of laboratory-confirmed COVID-19 more than 14 days before admission (postinfection patients).

Despite negative admission tests on admission, five inpatients developed symptomatic COVID-19 after admission. The details of these five cases (Patients A, B, C, D and E) who developed symptoms after admission are as follows: Patient A was an index case who transmitted the virus to another case in the same room, Patient B. This was nosocomial transmission between Patients A and B. Related to Patients A and B, three healthcare workers tested positive on DNA amplification tests. Although a mandatory surgical mask policy was in place and complied with by all medical staff and almost all inpatients, Patients A and B could not wear surgical masks due to respiratory dysfunction. Despite Patients A and B not being able to wear surgical masks, the medical staff caring for these individuals did not wear eye protection. Patients C, D and E were confirmed as other COVID-19 cases that developed after admission, which means they were sporadic cases as no additional clinically confirmed cases of nosocomial transmission were found between patients and/or healthcare workers.

Another two patients, who were admitted for a few days for gastrointestinal endoscopy, developed symptoms and tested positive at home within 48 h of discharge. Although patients do not generally receive a follow-up call and are not generally subjected to systematic testing after discharge, the patients reported that they developed symptoms and tested positive. These additional cases raised the possibility of transmission to other patients, and contact tracings were performed. However, laboratory-confirmed symptomatic COVID-19 was not reported in any other patients other than Patients A–E.

**Figure 1:**
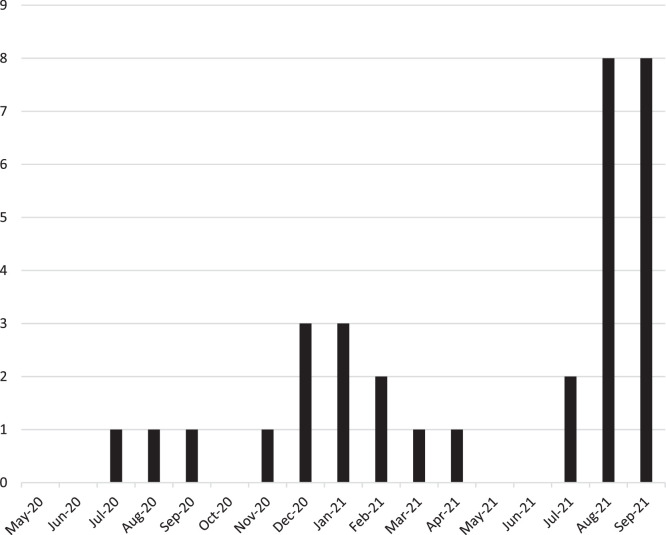
Monthly numbers of nucleic acid amplification test (NAAT)-positive cases in asymptomatic patients on admission during the study period. The Y-axis shows the number of NAAT-positive cases.

The number and time-series data of asymptomatic positive DNA amplification cases per month are shown in Figure 1. In both August and September 2021, the number of NAAT-positive cases exceeded eight, and positive case numbers ranged from 0 to 2 until July 2021. Even the maximum monthly positive rate remained at 0.52% in September 2021, whereas the minimum monthly positive rate was 0% in April, May, June and October 2020, and May and June 2021. Two positive cases were reported in August 2021, and three positive cases were reported in September 2021; all five cases were patients who had been diagnosed and treated elsewhere but were still testing positive at the time of admission to TMUH.

**Figure 2:**
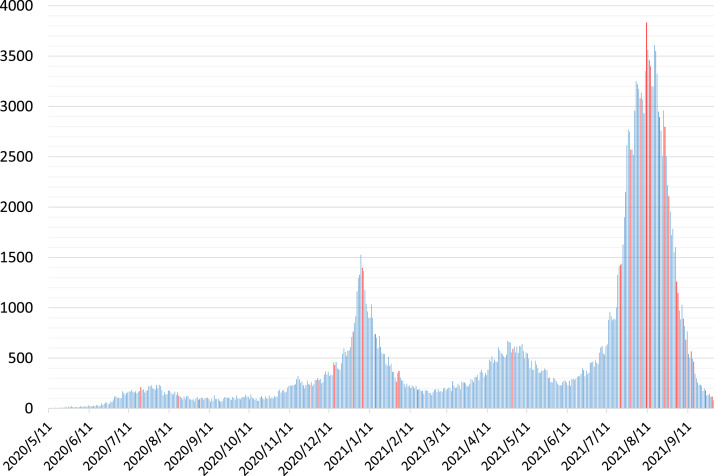
Notification trend of cases of coronavirus disease 2019 (COVID-19) in the Tokyo metropolitan area, and notifications by date of positive admission screening. The Y-axis shows the number of notified cases of COVID-19 in the Tokyo metropolitan area. The red bars represent the number of notified cases in the Tokyo metropolitan area when NAAT-positive cases at admission were confirmed at Tokyo Medical University Hospital.

The occurrence of positive results was almost concomitant with the general population epidemic trends (Figure 2). Based on COVID-19 notifications for the Tokyo metropolitan area during the same period, the minimum number of 92 reported notifications of positive admission screenings corresponded to a weekly average of 8.93 per 100,000 population in the Tokyo metropolitan area. Additionally, with higher notifications of COVID-19 in the Tokyo metropolitan area, the interval between positive cases became shorter, meaning more frequent positive results.

## Discussion

Previous studies of universal admission screening have reported positive rates ranging from 0.03% to 14.5%, depending on the study period and the epidemic status of the study site (Table 2). Epidemic surges requiring lockdowns have naturally increased the number of positive DNA amplification admission screenings, such as during March and April 2020 in New York City. However, many previous studies have reported low positive rates at admission among asymptomatic patients, at less than 1%, highlighting issues of cost-effectiveness. The positive rate data from New York City were too large and seemingly cannot be applied to other settings. The present study included the largest dataset to date, showing changes in rates during several epidemic surges, indicating that the low positive rate is valid and reasonable. At the same time, issues of cost-effectiveness should be considered in light of the low positive rate.

**Table 2:** Literature review of universal admission screening for asymptomatic pregnant women and other asymptomatic patients.

Country	Study period	Population	Positive result (rate)
Japan [2]	May–Sep 2020	Asymptomatic patients	2/6224 (0.03%)
Japan [3]	Aug 2020–May 2021	Asymptomatic patients	9/12,133 (0.07%)
Switzerland [7]	Apr 2020	Asymptomatic patients	8/2278 (0.04%)
Switzerland [18]	Apr–Jun 2020	Asymptomatic patients	6/4050 (0.15%)
Israel [8]	Sep–Dec 2020	Asymptomatic patients	133/8518 (1.56%)
Canada [1]	Apr–May 2020	Asymptomatic pregnant women	3/392 (0.77%)
UK [19]	May–June 2020	Asymptomatic pregnant women	1/457 (0.22%)
Japan [4]	Apr 2020	Asymptomatic pregnant women	2/52 (3.85%)
USA [20]	Apr 2020	Asymptomatic pregnant women	8/318 (2.52%)
USA [21]	Mar–Apr 2020	Asymptomatic pregnant women	29/211 (13.74%)
USA [22]	Mar–Apr 2020	Asymptomatic pregnant women	1/170 (0.5%)
USA [23]	Apr–May 2020	Asymptomatic pregnant women	17/3923 (0.43%)
Spain [6]	Apr–May 2020	Asymptomatic pregnant women	1/203 (0.49%)
Italy [24]	Mar–Apr 2020	Asymptomatic pregnant women	3/533 (0.56%)
USA [25]	Mar–Apr 2020	Asymptomatic pregnant women	21/124 (14.48%)
Present study	May 2020–Sep 2021	Asymptomatic patients	32/29,558 (0.11%)

The patient population that should be prioritized for infection prevention for nosocomial transmission appears to be presymptomatic patients. Previous studies have illustrated that presymptomatic patients, who can have a huge impact on transmission [10,11], comprised approximately 15% of asymptomatic patients at admission. On the other hand, the proportion of transmissions from totally asymptomatic patients remains unclear [12]. Surprisingly, a previous study found no secondary transmission from totally asymptomatic patients [10]. Clarifying the role of totally asymptomatic patients in transmission could increase the specific value of test screening for these patients.

A simulation study indicated that routine testing, such as more than twice per week, would be effective and would prevent transmission in high-risk settings, such as healthcare facilities [13]. Another study similarly reported that weekly molecular screening can play an important role in the prevention of transmission [14]. This evidence suggests that a single administration of NAAT screening is insufficient. In the present study, retests were not performed due to testing capacity and cost. This means that asymptomatic patients may not have been detected. On the other hand, some previous reports have noted that appropriate personal protective equipment and transmission precautions could minimize transmission, even in underdiagnosed situations [15,16]. Therefore, in addition to testing-based strategies, appropriate infection precaution strategies, focusing particularly on asymptomatic patients, should be adopted. A previous study from Korea reported that four cases with negative NAATs before admission and positive NAATs after admission resulted in a total of 18 additional cases [9]. Another study in a perioperative setting reported that five new positive cases occurred after surgery, resulting in one case of transmission [17]. In the present study, five asymptomatic cases developed laboratory-confirmed symptomatic COVID-19 based on more than 30,000 tests, whereas there was only one case of nosocomial transmission among patients with recognized COVID-19. This suggests the effectiveness of enhanced infection precaution strategies, as some research has suggested that minimal use of adequate personal protective equipment prevents transmission to healthcare workers [15], [16], [17]. However, it is important to note that nosocomial transmission by asymptomatic patients is difficult to detect.

Another negative aspect of highly test-dependent strategies is the detection of postinfectious status [9,17]. Epidemic surges also produced postinfected patients, and some continue to be long-term RNA shedders, who are misdiagnosed and detected by uniform screening testing. The five postinfection cases in the present study postponed admission and surgery despite their non-contagious status, representing negative aspects of universal admission DNA amplification test screening.

The criteria for the adaptation and withdrawal of test admission screening at each hospital remain an unsolved problem. The present study indicated fluctuating positive cases per month from 0% to 0.52%, almost coinciding with the prevalence in the community. The minimum number of COVID-19 daily notifications was 92 cases, and the weekly average was 8.93 per 100,000 population in the Tokyo metropolitan area among dates with positive NAAT admission screening. A Swiss study reported that universal admission testing did not identify a substantial number of asymptomatic cases in a low-prevalence setting (only 2.7 cases per 100,000 were reported) [7]. A similar result was indicated in a Korean study [9], where less than 20 cases per million were notified. Although adequate criteria for notification numbers are controversial, these numbers in the community may be helpful to judge the need for admission NAAT screening based on a medical recourse or testing capacity.

This study has some limitations. First, although admission test screening was performed comprehensively, follow-up DNA amplification screening (retesting), such as 4–7 days after admission, was not performed. This may result in underdetected transmission of COVID-19. However, during the 17 months of this study, no clinically confirmed nosocomial clusters of COVID-19 from underdetected asymptomatic or presymptomatic patients, other than the case recognized, were observed. Second, the effect of COVID-19 vaccination coverage was not included in the analysis of this study. The vaccination status of the study population could affect the effectiveness of pre-admission screening and the enhanced infection control strategies. Third, the effect of the virus variant was not analysed. In the future, changes in transmissibility or clinical symptoms of the virus due to its variants may influence evaluation of the effect of admission test screening. Fourth, serological testing and whole-genome screening were not performed. Test-based judgement and clinical case identification may result in a low estimation of SARS-CoV-2 infectious status because some asymptomatic or mildly symptomatic cases without further DNA amplification testing may develop after admission. Combining serological tests and whole-genome screening can reinforce the data. If whole-genome screening is undertaken, this may be able to detect and validate nosocomial transmission events. Fifth, the study was observational without a comparison group to measure the impact of this intervention. Determination of the effectiveness of admission test screening or combined infection prevention strategies requires further comparative study. Finally, the study results are from the prevalence and surge levels in Japan, and may not apply to other countries and phases. The data and effects of universal admission screening have varied depending on the prevalence level.

## Conclusions

More than 30,000 DNA amplification pre-admission screening tests detected only 32 cases of asymptomatic or presymptomatic COVID-19. Although five of these cases developed symptomatic COVID-19, enhanced infection control precaution strategies in addition to universal admission screening may have reduced the occurrence of clusters.

## Funding

None.

## Author contributions

IN: study design, data analysis, drafting the manuscript.

YW: data collection, critical revision of the manuscript.

HF: data collection, critical revision of the manuscript.

TK: critical revision of the manuscript.

HW: study design.

TI: study design, data analysis, drafting the manuscript.

## Ethical approval

This study was approved by the Ethical Committee of Tokyo Medical University (Approval No. T2020-0161). The need for consent was formally waived by the Ethical Committee of Tokyo Medical University. This study was performed in accordance with the 1964 Declaration of Helsinki and its later amendments.

## Availability of data and materials

The dataset supporting the conclusions of this article is included in the article.

## Conflict of interest statement

None declared.
